# Crystal structures and Hirshfeld surface analyses of (*E*)-*N*′-benzyl­idene-2-oxo-2*H*-chromene-3-carbo­hydrazide and the disordered hemi-DMSO solvate of (*E*)-2-oxo-*N*′-(3,4,5-trimeth­oxybenzyl­idene)-2*H*-chromene-3-carbohydrazide: lattice energy and inter­molecular inter­action energy calculations for the former

**DOI:** 10.1107/S2056989019012015

**Published:** 2019-09-03

**Authors:** Ligia R. Gomes, John Nicolson Low, James L. Wardell, Camiola Capelini, Vitoria R.F. Câmara, Edson F. da Silva, Samir A. Carvalho

**Affiliations:** a REQUIMTE, Departamento de Química e Bioquímica, Faculdade de Ciências da Universidade do Porto, Rua do Campo Alegre, 687, P-4169-007, Porto, Portugal; b FP-ENAS-Faculdade de Ciências de Saúde, Escola Superior de Saúde da UFP, Universidade Fernando Pessoa, Rua Carlos da Maia, 296, P-4200-150 Porto, Portugal; c Department of Chemistry, University of Aberdeen, Meston Walk, Old Aberdeen, AB24 3UE, Scotland; d Instituto de Tecnologia em Fármacos - Farmanguinhos, Fundaçâo Oswaldo Cruz, 21041-250 Rio de Janeiro, RJ, Brazil; e Escola de Ciéncia e Tecnologia - ECT, Universidade do, Grande Rio - Unigranrio, 25071-202, Duque de Caxias, RJ, Brazil

**Keywords:** crystal structure, tuberculosis, Hirshfeld surface analysis, nitro­gen-containing 2-oxo-2*H*-chromene derivative

## Abstract

The crystal structures of the disordered hemi-DMSO solvate of (*E*)-2-oxo-*N*′-(3,4,5-tri­meth­oxy­benzyl­idene)-2*H*-chromene-3-carbohydrazide, C_20_H_18_N_2_O_6_·0.5C_2_H_6_OS, and (*E*)-*N*′-benzyl­idene-2-oxo-2*H*-chromene-3-carbohydrazide, C_17_H_12_N_2_O_3_ (**4**: *R* = C_6_H_5_), are discussed. The non-hydrogen atoms in compound [**4**: *R* = (3,4,5-MeO)_3_C_6_H_2_)] exhibit a distinct curvature, while those in compound, (**4**: *R* = C_6_H_5_), are essential coplanar. In (**4**: *R* = C_6_H_5_), C—H⋯O and π–π intra­molecular inter­actions combine to form a three-dimensional array. A three-dimensional array is also found for the hemi-DMSO solvate of [**4**: *R* = (3,4,5-MeO)_3_C_6_H_2_], in which the mol­ecules of coumarin are linked by C—H⋯O and C—H⋯π inter­actions, and form tubes into which the DMSO mol­ecules are cocooned. Hirshfeld surface analyses of both compounds are reported, as are the lattice energy and inter­molecular inter­action energy calculations of compound (**4**: *R* = C_6_H_5_).

## Abstract 2

The structures and Hirshfeld surface analyses of the title compounds are reported.

## Chemical context   

Tuberculosis (TB) is one of the world’s most infectious killer diseases, claiming 4,500 lives each day (https://www.who.int/en/news-room/fact-sheets/detail/tuberculosis). The development of drug resistance to the first-line drugs seriously compounds the dangers of the disease. The latest multidrug-resistant TB data analysis shows that 4.1% of new and 19% of previously treated TB cases in the world are estimated to have rifampicin- or multidrug-resistant tuberculosis (MDR/RR-TB) and about 6.2% of the MDR-TB cases have additional drug resistance, extensively drug-resistant TB (XDR-TB) (www.who.int/tb/challenges/mdr/MDR-RR_TB_factsheet_2017.pdf). As a result of the increase of MDR-TB/XDR-TB and AIDS cases worldwide, associated with the lack of efficacy of available drugs, the discovery of new potent and safer drug-candidate prototypes able to treat this disease has become an urgent challenge.

The *N*-acyl­hydrazone functional group, –C(O)—NH—N=CH–, is found in many compounds having important and diverse biological activities (Fraga & Barreiro, 2006 ▸; Singh *et al.*, 2016 ▸), including their use in the fight against tuberculosis, especially the drug-resistant forms (Cardoso *et al.*, 2011 ▸; Souza *et al.*, 2017 ▸). Specifically, *N*-acyl­hydrazonyl-containing 2*H*-chromene derivatives have been found to possess significant anti-mycobacterial activities (Angelova *et al.*, 2017 ▸; Cardoso *et al.*, 2011 ▸). The Angelova *et al.* (2017 ▸) study revealed compounds of type **1**–**3** (*R* = ar­yl) in the schematic diagram as having *in vitro* anti­mycobacterial activities against *Mycobacterium tuberculosis H37Rv* comparable to the first-line drugs, isoniazid (INH) and ethambutol, while the Cardoso *et al.* (2011 ▸) study indicated compounds of type **4** (*R* = ar­yl) in the schematic diagram to be active against *Mycobacterium* tuberculosis ATCC 27294. Of inter­est, **4** (*R* = 3-MeOC_6_H_4_) and (*R* = 4-MeOC_6_H_4_), but not **4** [*R* = 3,4-(MeO)_2_C_6_H_3_] exhibited better activities than did pyrazinamide (Cardoso *et al.*, 2011 ▸).

We have continued studies of the *Mycobacterial* activities of compounds of type **4** (Capelini *et al.*, 2019 ▸) against various strains, namely *M. tuberculosis* H37Rv ATCC 27294 INH-resistant *Mtb*, multidrug-resistant *Mtb* and wild INH/RIF-resistant *Mtb* isolates: [**4**: *R* = (3,4,5-MeO)_3_C_6_H_2_] exhibited significant activity against the INH resistant/RIP resistant strain, *M. tuberculosis* SR 5110/1116. We now wish to report the crystal structures and the Hirshfeld surface analyses of a DMSO hemi-solvate of this compound and also that of the parent compound, (**4**: *R* = C_6_H_5_), an inactive compound. In addition, lattice energy and inter­molecular inter­action energy calculations are reported for **4** (*R* = C_6_H_5_). This article also continues our reporting of the structures of nitro­gen-containing 2-oxo-2*H*-chromene derivatives (Gomes *et al.*, 2016*a* ▸).
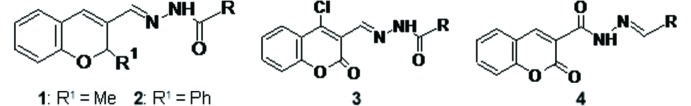


## Structural commentary   

**Figure 1:**
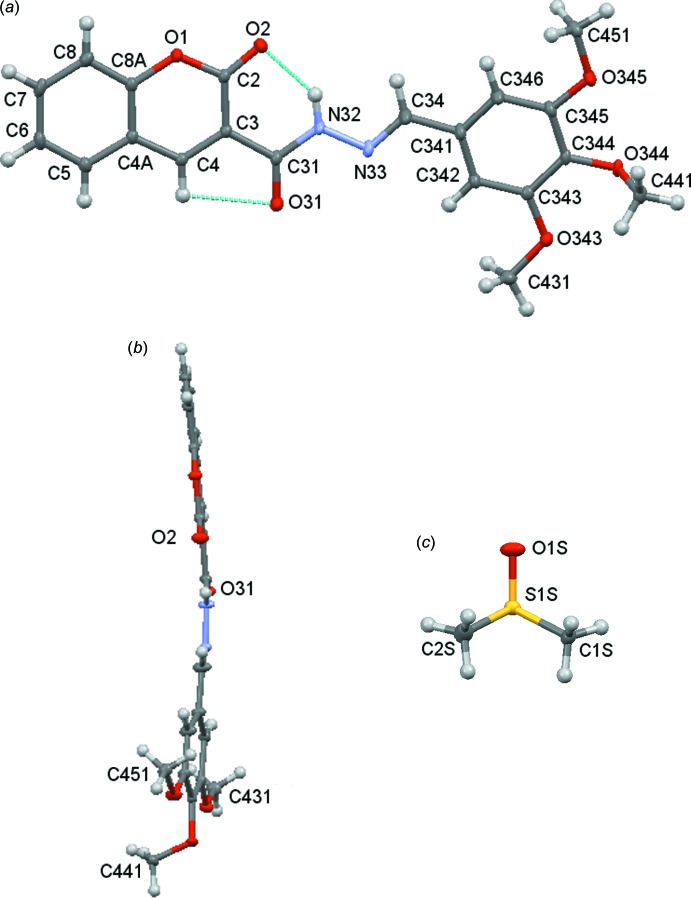
Compound [**4**: *R* = 3,4,5-(MeO)_3_C_6_H_2_·0.5DMSO]. (*a*) Mol­ecular structure and numbering scheme for [**4**: *R* = 3,4,5-(MeO)_3_C_6_H_2_] with displacement ellipsoids drawn at the 50% level, (*b*) side-on view of the conformation of [**4**: *R* = 3,4,5-(MeO)_3_C_6_H_2_] and (*c*) the DMSO hemi-solvate showing one component of disorder.

**Figure 2:**
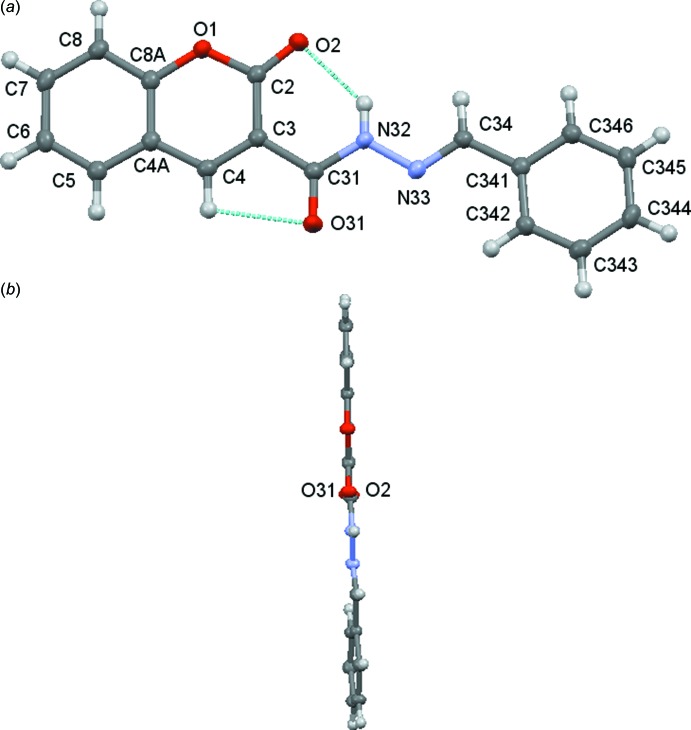
Compound (**4**: *R* = C_6_H_5_). (*a*) Mol­ecular structure and numbering scheme with displacement ellipsoids drawn at the 50% level and (*b*) side-on view of the conformation.

**Figure 3:**
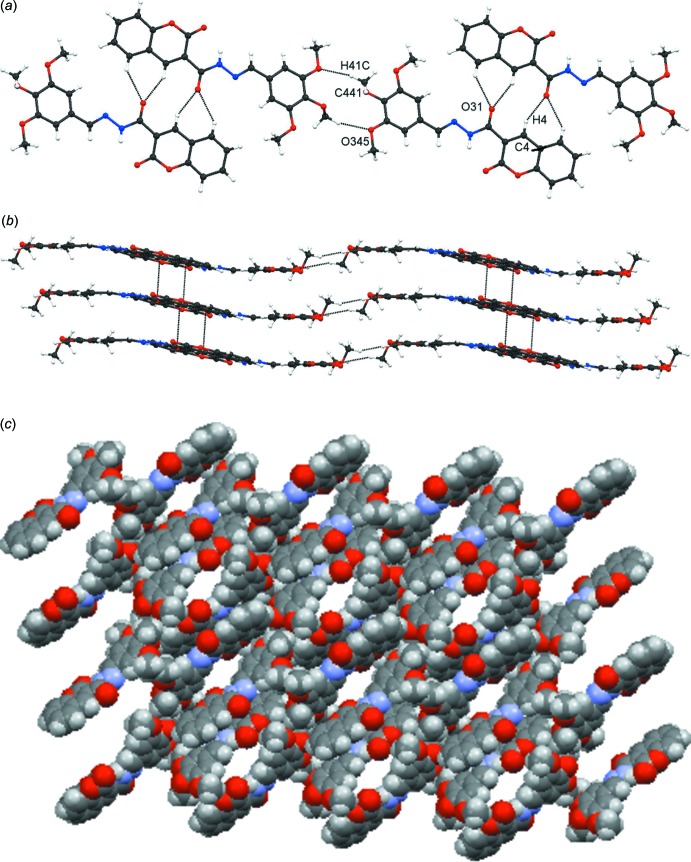
Compound [**4**: *R* = 3,4,5-(MeO)_3_C_6_H_2_·0.5DMSO]. (*a*) A two-mol­ecule-wide column of mol­ecules, formed from C4—H4⋯O31, C5—H5⋯O31 and C441—H41*C*⋯O345 hydrogen bonds, (*b*) columns linked into undulating sheets by C31=O31⋯π(1) inter­actions and (*c*) a spiral of mol­ecules, which creates a channel into which the disordered solvate mol­ecules are held by a number of C—H⋯X (*X* = O, N or S) hydrogen bonds: the channel is generated from C431–H43*B*⋯π(3), C451—H51*B*⋯π(3) and C451—H51*C*⋯π(2) inter­actions and lies along the crystallographic twofold axis.

The solvate [**4**: *R* = (3,4,5-MeO)_3_C_6_H_2_·0.5DMSO] crystallizes in the ortho­rhom­bic space group *C*2/*c*, with one mol­ecule of the coumarin and with a half DMSO solvate mol­ecule spread over two symmetry-related sites in the asymmetric unit, Fig. 1 ▸. Compound (**4**: *R* = C_6_H_5_) crystallizes in the triclinic space group *P* 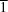 with one mol­ecule in the asymmetric unit, see Fig. 2 ▸. The geometry about the C=N bond of the hydrazine moiety is (*E*) in both cases. There are intra­molecular C2—H2⋯O1 and C4—H4⋯O31 hydrogen bonds (Tables 2 ▸ and 3 ▸) present in both mol­ecules. The non-hydrogen atoms, with the additional exclusion of atoms in the three meth­oxy groups in the phenyl substituent unit of [**4**: *R* = (3,4,5-MeO)_3_C_6_H_2_·0.5DMSO], form a distinctively curved arrangement, as illustrated in Fig. 1 ▸ *b*. In contrast, the non-hydrogen atoms in (**4**: *R* = C_6_H_5_) are essentially co-planar, see Fig. 3 ▸. The bond lengths in the linker chain between the coumarin and phenyl moieties are indicative of electronic delocalization, see Table 1 ▸.The inter­planar angles, coumarin/linker, linker/phenyl and phen­yl/coumarin in [**4**: *R* = (3,4,5-MeO)_3_C_6_H_2_·0.5DMSO], are 7.70 (7), 11.43 (8) and 14.97 (5)°, compared to 2.89 (5), 5.07 (5) and 7.05 (4)°, respectively, in (**4**: *R* = C_6_H_5_). In [**4**: *R* = (3,4,5-MeO)_3_C_6_H_2_], as expected for a compound with adjacent meth­oxy groups on the 3,4 and 5 positions of a phenyl ring, the middle meth­oxy group is out of the plane of its phenyl group (see, for example, Peralta *et al.*, 2007 ▸; Howie *et al.*, 2010 ▸; Gomes *et al.*, 2016*b* ▸).

## Supra­molecular features   

### Inter­molecular inter­actions   

There are no classical inter­molecular O—H⋯*X* (*X* = O or N) in the crystal of [**4**: *R* = (3,4,5-MeO)_3_C_6_H_2_·0.5DMSO]: the mol­ecules of [**4**: *R* = (3,4,5-MeO)_3_C_6_H_2_] are linked by a number of C—H⋯O and C—H⋯π hydrogen bonds (Table 3 ▸) and by a C=O⋯π(1) inter­action: the three rings in compounds **4** have been given the designations π(1) for the O1/C2–C4/C4*A*/C8*A*, π(2) for the C4*A*/C5–C8/C8*A* and π(3) for the C341–C346 rings with centroids *Cg*1, *Cg*2 and *Cg*3, respectively. A two-mol­ecule wide column is generated from a combination of the C4—H4⋯O31, C5—C5⋯O31 and C441—H41*C*⋯O345 hydrogen bonds, see Fig. 3 ▸ *a*. Within the columns, the C4—H4⋯O31 and C5—H5⋯O31 inter­actions generate 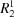
(5) rings and pairs of the C441—H41*C*⋯O345 hydrogen bonds lead to 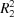
(12) rings. These two-mol­ecule-wide columns are linked by the carbon­yl–arene inter­action C31=O31⋯π(1) into undulating sheets, see Fig. 3 ▸ *b*. A further structural subset is formed from a series of C—H⋯π inter­actions: C431—H43*B*⋯π(3) and C451—H51*B*⋯π(3) separately form chains of [**4**: *R* = (3,4,5-MeO)_3_C_6_H_2_] propagating in the *b-*axis direction, while the C451—H51*C*⋯π2 inter­action generates a spiral chain of mol­ecules; together these three inter­actions form a tube, into which the disordered DMSO mol­ecule is cocooned, held there by a number of C—H⋯*X* (*X* = O, N and S) hydrogen bonds. A view of the channels in which the the disordered DMSO sits is shown in Fig. 3 ▸ *c*. These channels run along the crystallographic twofold axis.

**Figure 4:**
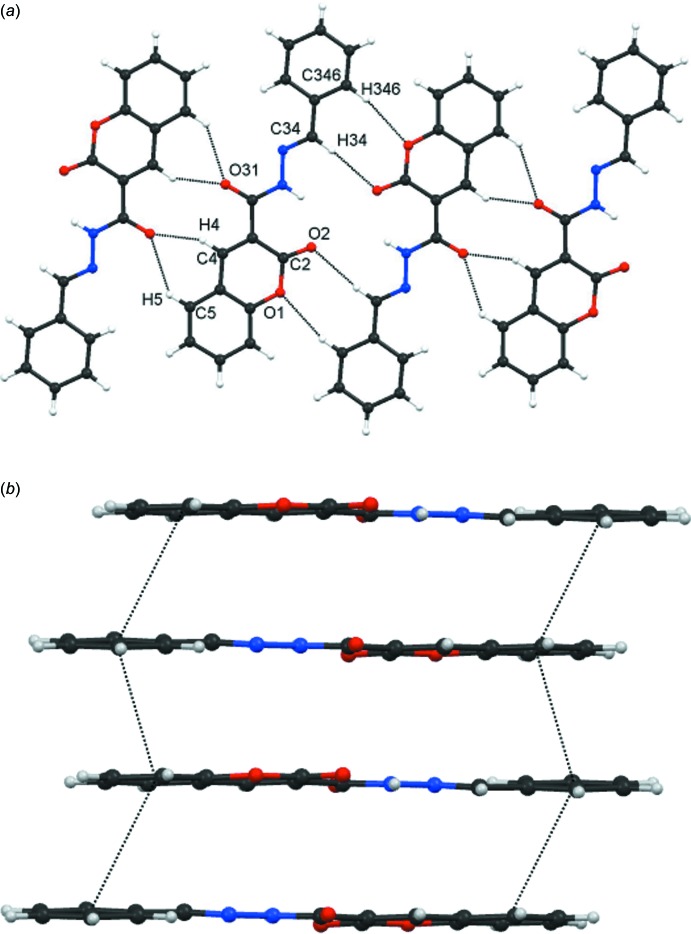
Compound (**4**: *R* = C_6_H_5_). (*a*) Part of a one-mol­ecule-wide column formed from linking mol­ecules by a combination of C4—H4⋯O31, and C5—H5⋯O31, C34—H34⋯O2 and C346—H346⋯O1 hydrogen bonds. Within these columns are 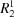
(5), 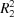
(10) and 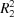
(16) rings and (*b*) part of a column formed from two alternating π–π^i^ and π–π^ii^ inter­actions [symmetry codes: (i) 1 − *x*, −*y*, 1 − *z*; (ii) 1 − *x*, 1 − *y*, 1 − *z*].

The inter­molecular inter­actions in compound (**4**: *R* = C_6_H_5_) are C—H⋯O hydrogen bonds, see Table 3 ▸, and π–π stacking inter­actions. Symmetric dimers are formed from pairs of each of C4—H4⋯O31 and C5—H5⋯O31, see Fig. 4 ▸ *a*. Within the dimers are two 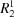
(5) and one 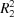
(10) rings. These dimers are then linked by pairs of C34—H34⋯O2 and C346—H346⋯O1 hydrogen bonds into a one-mol­ecule-wide column, generating two 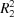
(8) and one 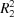
(16) rings. A second sub-structure is formed from alternating π–π^i^ and π–π^ii^ inter­actions, involving the C4*A/*C5–C8/C8*A* ring with centroid *Cg*2 and the C341–C346 ring with centroid *Cg*3, see Fig. 4 ▸ *b* [symmetry codes: (i) 1 − *x*, −*y*, 1 − *z*; (ii) 1 − *x*, 1 − *y*, 1 − *z*]*.* The π–π^i^ inter­action is considered to be the stronger, both from the *Cg*⋯*Cg* separation [3.8417 (6) compared to 4.1750 (6) Å] and from its greater π overlap, average slippages being 1.820 and 2.325 Å (the rings are inclined to each other). Further confirmation of the relative importance of the two inter­actions comes from the energy calculations, see Section 3.3. The combination of all the inter­molecular inter­actions provides a three-dimensional arrangement.

### Hirshfeld Surface analyses   

**Figure 5:**
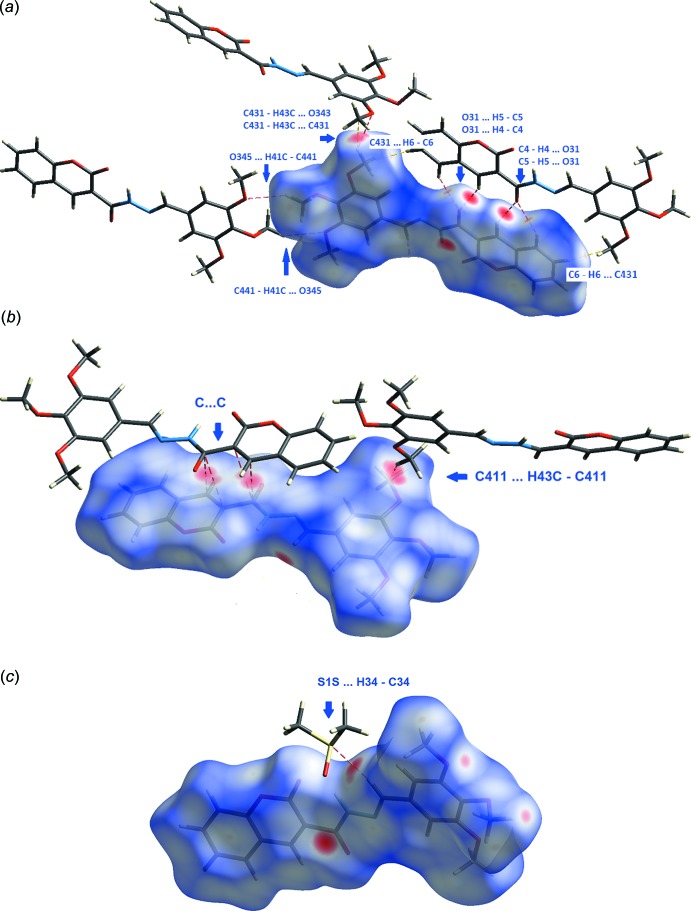
Hirshfeld surface views for [**4**: *R* = 3,4,5-(MeO)_3_C_6_H_2_·0.5DMSO]. The red areas on the surfaces correspond to close contacts. In (*a*) the site of a close H6⋯C431 contract is indicated: H6⋯C431^i^ = 2.85 Å (sum of contact radii = 2.90 Å) [symmetry code: (i) 1 − *x*, −*y*, 1 − *z*].

**Figure 6:**
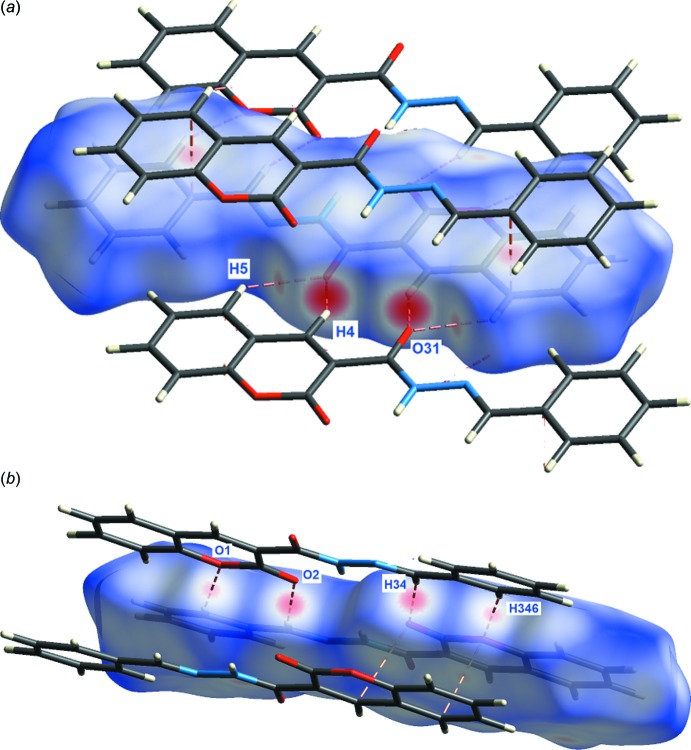
Two views of the Hirshfeld surface of (**4**: *R* = C_6_H_5_)·The red areas on the surfaces correspond to the designated close contacts.

**Figure 7:**
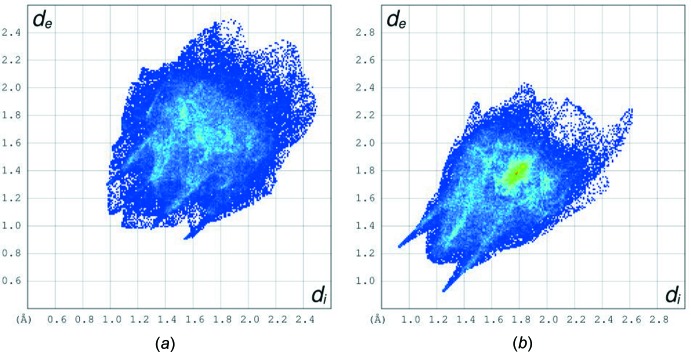
FP plots for (*a*) [**4**: *R* = 3,4,5-(MeO)_3_C_6_H_2_·0.5DMSO] and (*b*) (**4**: *R* = C_6_H_5_) in which the blue spikes ending at (1.2; 0.9) and (0.9;1.1) relate to O⋯H/H⋯O contacts and the high intensity of pixels, green and red areas relate to C⋯C contacts.

Hirshfeld surfaces (Spackman & Jayatilaka, 2009 ▸) and two-dimensional fingerprint (FP) plots (Spackman & McKinnon, 2002 ▸), provide complementary information concerning the inter­molecular inter­actions discussed above. The analyses were generated using *CrystalExplorer3.1* (Wolff *et al.*, 2012 ▸). The Hirshfeld surfaces mapped over *d*_norm_ were scaled between −0.33 and 1.23, and are shown in Fig. 5 ▸ for [**4**: *R* = (3,4,5-MeO)_3_C_6_H_2_·0.5DMSO] and in Fig. 6 ▸ for (**4**: *R* = C_6_H_5_). The red areas on the surfaces correspond to close contacts, and have been designated. The FP plots for [**4**: *R* = (3,4,5-MeO)_3_C_6_H_2_·0.5(DMSO)] and (**4**: *R* = C_6_H_5_) are shown in Fig. 7 ▸ *a* and 7*b*, respectively. The blue spikes in the FP plot for (**4**: *R* = C_6_H_5_) ending at (1.2; 0.9) and (0.9;1.1) relate to O⋯H/H⋯O contacts and the high intensity of pixels, green and red areas relate to C⋯C contacts.

The percentages of atom⋯atom close contacts are listed in Table 4 ▸. Leaving the H⋯H contacts aside, the highest percentages of atom⋯atom close contacts for [**4**: *R* = (3,4,5-MeO)_3_C_6_H_2_·0.5DMSO], are 28.4 and 23.7% for H⋯O/O⋯H and H⋯C/C⋯H, respectively. The corresponding values for (**4**: *R* = C_6_H_5_) are 20.2 and 17.9%.

### Lattice energy and inter­molecular inter­action energy calculations   

Lattice energies and inter­molecular inter­action energies were calculated using the PIXEL routine implemented in the *CLP* package (Gavezzotti, 2003 ▸, 2008 ▸) which allows the calculation of inter­molecular energies by distributed charge description on the basis of a preliminary evaluation of charge density from *GAUSSIAN* at the MP2/6-311G** level of theory (CUBE option). The PIXEL mode calculates the total stabilization energies of the crystal packing, *E*_tot_, distributed as coulombic, (*E*_coul_), polarization (*E*_pol_), dispersion (*E*_disp_) and repulsion (*E*_rep_) terms between separate, rigid mol­ecules. Coulombic terms are treated on the basis of Coulombic law, polarization terms are calculated as a linear dipole approximation, dispersion terms are based on London’s inverse six-power approximation involving ionization potentials and polarizabilities and the repulsion term comes from a modulated function of the wave-function overlap.

**Figure 8:**
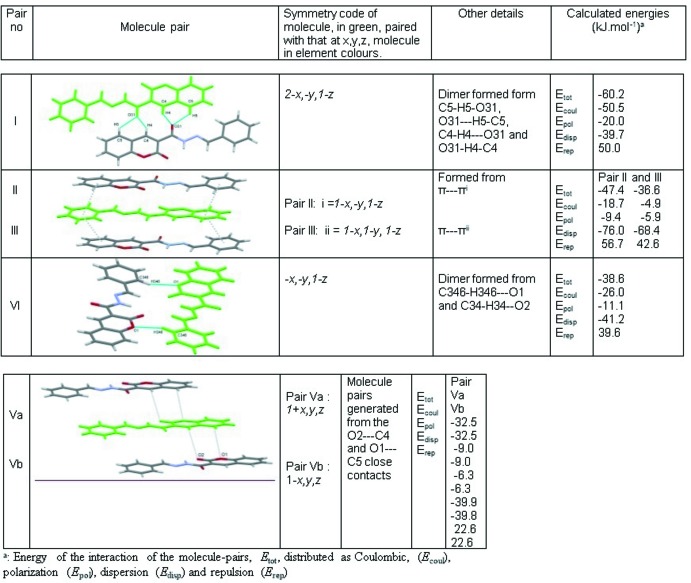
Calculated energies for the most significant mol­ecule pairs in (**4**: *R* = C_6_H_5_).

The presence of a half mol­ecule of DMSO lying at a symmetry centre in [**4**: *R* = (3,4,5-MeO)_3_C_6_H_2_·0.5DMSO], precludes the PIXEL analysis for this structure. Partial analysis of the PIXEL calculations, however, was carried out on (**4**: *R* = C_6_H_5_). The six mol­ecule pairs that contribute most to the total energy of the packing of (**4**: *R* = C_6_H_5_) are shown in Fig. 8 ▸.

The various energies for these six significant mol­ecule pairs are also listed in Fig. 8 ▸. As such energy values pertain to both the reference mol­ecule at *x*, *y*, *z* and its partner in the mol­ecule pair, the energies thus associated with the reference mol­ecule at *x*, *y*, *z* are half of these sums. The total PIXEL energy calculated for the complete lattice is −157.9 kJ.mol^−1^. Of that, −123.9 kJ.mol^−1^(78.5%) is derived from the six mol­ecule pairs shown in Fig. 8 ▸. The percentage contribution of pairs involved in O—H⋯O hydrogen bonds is 29.4% while pairs making C⋯C close contacts contribute 26.6% to the total stabilization energy.

## Database survey   

A search of the Cambridge Structural Database (CSD Version 5.39, August 2018 update; Groom *et al.*, 2016 ▸) found only one structure of type **4**, namely *R* = 4-MeOC_6_H_4_), which is currently undergoing enhancement with a current *R* value of 0.094 (Low & Wardell, 2019 ▸) and was briefly mentioned in a submitted article (Capelini *et al.*, 2019 ▸). The mol­ecule of (**4**: *R* = 4-MeOC_6_H_4_) has a near-planar conformation and possesses equivalent intra­molecular hydrogen bonds to those shown by the compounds reported in this article. A database search revealed other types of nitro­gen-containing 2-oxo-2*H*-chromene derivatives, including amido derivatives (Gomes *et al.*, 2016*a* ▸,*b* ▸); see also: DOLYEK (Borges *et al.*, 2014*a* ▸), DOLYIO (Cagide *et al.*, 2015 ▸) and DOLYOU (Borges *et al.*, 2014*b* ▸, 2016 ▸). Angelova *et al.* (2017 ▸) reported the structures of (**1**: *R* ^1^ = Me, *R* = C_6_H_5_) and (**1**: R^1^ = Me, *R* = pyridine-4-yl).

## Synthesis and crystallization   

### General procedure for the synthesis of compounds 4   

To a suspension of coumarinic acid (*cis*-*o*-hydroxycinammic acid, C_9_H_3_OH) (29 mmol, 1.0 equiv.) in CH_3_CN (100 ml) at room temperature, was added HOBt (34.64 mmol, 1.2 equiv.), followed by EDC (65.40 mmol, 2.25 equiv). The reaction was stirred at room temperature for 2 h, and slowly added to a solution of hydrazine hydrate (58.20 mmol, 2.0 equiv.) in CH_3_CN (100 mL) maintaining the temperature below 283 K. Water (70ml) was added to the reaction mixture, which was extracted successively with chloro­form (3 × 95 mL) and aqueous 5% sodium bicarbonate (3 × 120 mL). The organic phases were collected and rotary evaporated to yield the coumarinic hydrazide (**5**), as a yellow solid. Crystallization of compound [**4**: *R* = (3,4,5-(MeO)_3_C_6_H_2_] from DMSO solution produced the hemi-DMSO solvate, which on heating slowly decomposed to a dark residue. Attempts to gain suitable crystals for the structural study by slow recrystallization from ethanol solution at room temperature failed.

**(** ***E*** **)-** ***N*** **’-Benzyl­idene-2-oxo-2** ***H*** **-chromene-3-carbohydrazide (4:** ***R*** **= C_6_H_5_)**. Yield: 78%. m.p. 403.7 K.

**^1^H NMR (400 MHz, DMSO-** ***d*** **_6_)** ***δ*** 7.48 (4H, *m*), 7.55 (1H, *d*, *J* = 8.32 Hz), 7.77 (3H, *m*), 8.02 [1H, *dd*, *J*(*o*) = 7.84 Hz, *J*(*m*) = 1.52 Hz], 8.47 (1H, *s*), 8.92 (1H, *s*) 11.76 (1H, *s*).

**^13^C NMR (100 MHz, DMSO-** ***d*** **_6_)** ***δ*** 116.2, 118.4, 119.3, 125.3, 127.4, 128.9, 130.3, 130.4, 133.9, 134.3, 147.8, 149.4, 153.9, 158.1, 159.8.

**EI/MS (** ***m*** **/** ***z*** **) [** ***M*** **+ Na]^+^**: 315.11.

**IR (KBr)** ***ν*** **_max_ cm^−1^**: 3216.34 (N—H, bonded), 3064.30 (C—H, *sp* ^2^), 1695.02 (C=O, lactone), 1663.15 (C=O, amide), 1604.10 (C=C, double bond coumarin), 1531.50 and 1488.69 (C=C, aromatic), 788 and 748 (monosubstituted aromatic).

To a solution of the coumarinic hydrazide (**5**) (0.98 mmol) in absolute ethanol (25 mL), containing a catalytic amount of 37% aq. hydro­chloric acid, were added 1.03 mmol (1.05 equiv) of the desired benzaldehyde derivative. The mixture was refluxed until TLC indicated the complete consumption of **5** and the precipitate was collected and dried to yield the desired compound **4**, in yields ranging from 55 to 84%.

**(** ***E*** **)-2-Oxo-** ***N*** **’-(3,4,5-tri­meth­oxy­benzyl­idene)-2** ***H*** **-chromene-3-carbohydrazide [4:** ***R*** **= (3,4,5-MeO)_3_C_6_H_2_]**. Yield: 76%. m.p. 368.7 K.

**^1^H NMR (400 MHz, DMSO-** ***d*** **_6_) δ** 3.72 (3H, *s*), 3.84 (6H, *s*), 7.08 (2H, *s*), 7.47 [1H, *t*, *J*(*o*) = 7.88 Hz, *J*(*m*) = 0.96 Hz], 7.55 [1H, *d*, *J*(*o*) = 8.36 Hz], 7.78 [1H, t, *J*(*o*) = 7.88 Hz, *J*(*m*) = 1.6 Hz], 8.02 [1H, *dd*, *J*(*o*) = 7.84 Hz, *J*(*m*) = 1.44 Hz], 8.38 (1H, *s*), 8.90 (1H, *s*), 11.74 (1H, *s*).

**^13^C NMR (100 MHz, DMSO-** ***d*** **_6_)δ** 55.8, 60.0, 104.5, 116.1, 118.3, 119.3, 125.2, 129.2, 130.2, 134.2, 139.4, 147.6, 149.3, 153.0, 153.8, 157.9, 159.8.

**EI/MS (** ***m*** **/** ***z*** **) [** ***M*** **+ H]^+^**: 383.13, **[** ***M*** **+ Na]^+^**: 405.09.

**IR (KBr)** ***ν*** **_max_ cm^−1^**: 3185.12 (N—H), 2941.30 (C—H, *sp* ^3^), 1698.89 (C=O, lactone), 1666.73 (C=O, amide), 1609.91 (C=C, double bond coumarin), 1532.56 and 1499.88 (C=C, aromatic), 1229.99 and 1121.84 (C—O—C).

Suitable crystals of **4** for the structural study were obtained by slow evaporation of a solution in ethanol at room temperature

## Refinement   

Crystal data, data collection and structure refinement details are summarized in Table 5 ▸. C-bound H atoms were refined as riding atoms at calculated positions [C—H = 0.95–0.98 Å with *U*_iso_(H) = 1.2–1.5*U*_eq_(C)]. That attached to the N atom was refined.

In [**4**: *R* = 3,4,5-MeO_3_C_6_H_2_·0.5DMSO] the solvent DMSO mol­ecule lies on a crystallographic twofold axis. It was refined with a fixed occupancy factor of 0.5. A refinement of the s.o.f. gave a value of 0.488. The DMSO mol­ecules are located in channels which run along the twofold axis.

## Supplementary Material

Crystal structure: contains datablock(s) I, II, global. DOI: 10.1107/S2056989019012015/lh5917sup1.cif

Structure factors: contains datablock(s) I. DOI: 10.1107/S2056989019012015/lh5917Isup2.hkl

Structure factors: contains datablock(s) II. DOI: 10.1107/S2056989019012015/lh5917IIsup3.hkl

Supporting information file. DOI: 10.1107/S2056989019012015/lh5917Isup4.cml

Supporting information file. DOI: 10.1107/S2056989019012015/lh5917IIsup5.cml

CCDC references: 1917252, 1917524

Additional supporting information:  crystallographic information; 3D view; checkCIF report

## Figures and Tables

**Table 1:** Selected bond lengths (Å) in the linker chain between the coumarin and phenyl moieties

Bond	[**4**: *R* = 3,4,5-MeO_3_C_6_H_2_·0.5DMSO]	(**4**: *R* = C_6_H_5_)
C2—O2	1.2133 (12)	1.2103 (11)
C31—O31	1.2234 (13)	1.2237 (12)
C3—C31	1.5056 (13)	1.5003 (13)
C31—N32	1.3543 (13)	1.3530 (13)
N32—N33	1.3793 (11)	1.3768 (11)
N33—C34	1.2753 (14)	1.2753 (13)
C34—C341	1.4629 (14)	1.4649 (13)

**Table 2:** Hydrogen-bond geometry (Å, °) for [**4**: *R* = (3,4,5-MeO)_3_C_6_H_2_·0.5DMSO][Chem scheme1] *Cg*1, *Cg*2 and *Cg*3 are the centroids of the O1/C2–C4/C4*A*/C8*A*, C4*A*/C5–C8/C8*A* and C341–C346 rings, respectively.

*D*—H⋯*A*	*D*—H	H⋯*A*	*D*⋯*A*	*D*—H⋯*A*
N32—H32⋯O2	0.870 (16)	1.955 (15)	2.6878 (12)	141.0 (14)
C441—H41*C*⋯O345^i^	0.98	2.58	3.4772 (12)	152
C451—H51*A*⋯O1^ii^	0.98	2.65	3.4463 (13)	138
C34—H34⋯O1*S*	0.95	2.57	3.30 (5)	134
C34—H34⋯O1*S* ^ii^	0.95	2.63	3.34 (5)	133
C34—H34⋯S1*S*	0.95	2.69	3.6158 (12)	166
C431—H43*C*⋯O343^iii^	0.98	2.50	3.2505 (13)	133
C2*S*—H2*SA*⋯N32^iv^	0.98	2.61	3.3000 (6)	127
C4—H4⋯O31	0.95	2.45	2.7761 (12)	100
C4—H4⋯O31^v^	0.95	2.38	3.2415 (12)	150
C5—H5⋯O31^v^	0.95	2.59	3.3931 (13)	143
C431—H43*B*⋯*Cg*3^vi^	0.98	2.73	3.5882 (13)	147
C451—H51*B*⋯*Cg*3^vi^	0.98	2.95	3.8562 (12)	155
C451—H51*C*⋯*Cg*2^vii^	0.98	2.83	3.6883 (13)	147
C31—O31⋯*Cg*1^vii^	0	0	3.3971 (6)	90 (1)

Symmetry codes: (i) 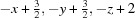; (ii) 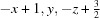; (iii) 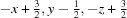; (iv) 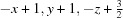; (v) 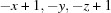; (vi) 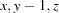; (vii) 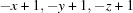.

**Table 3:** Hydrogen-bond geometry (Å, °) for (**4**: *R* = C_6_H_5_)

*D*—H⋯*A*	*D*—H	H⋯*A*	*D*⋯*A*	*D*—H⋯*A*
N32—H1⋯O2	0.857 (15)	2.062 (15)	2.7238 (10)	133.5 (12)
C34—H34⋯O2^i^	0.95	2.54	3.4417 (11)	159
C4—H4⋯O31	0.95	2.40	2.7415 (11)	101
C4—H4⋯O31^ii^	0.95	2.28	3.1377 (12)	149
C5—H5⋯O31^ii^	0.95	2.57	3.3456 (12)	139
C346—H346⋯O1^i^	0.95	2.63	3.5195 (11)	156

Symmetry codes: (i) 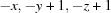; (ii) 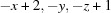.

**Table 4:** Percentages for atom⋯atom close contacts

Compound	[**4**: *R* = (3,4,5-MeO)_3_C_6_H_2_·0.5DMSO]	(**4**: *R* = C_6_H_5_)
O⋯H/H⋯O	20.2	28.4
O⋯N/N⋯O	1.9	–
O⋯C/C⋯O	6.0	2.4
O⋯O	–	1.2
N⋯C/C⋯N	3.3	2.3
N⋯H/H⋯N	2.4	2.7
H⋯C/C⋯H	17.9	23.7
C⋯C	8.9	1.7
H⋯H	39.2	37.1

**Table 5:** Experimental details

	[**4**: *R* = (3,4,5-MeO)_3_C_6_H_2_·0.5DMSO]	(**4**: *R* = C_6_H_5_)
Crystal data		
Chemical formula	C_20_H_18_N_2_O_6_·0.5C_2_H_6_OS	C_17_H_12_N_2_O_3_
*M*_r_	421.43	292.29
Crystal system, space group	Monoclinic, *C*2/*c*	Triclinic, *P* 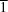
Temperature (K)	100	100
*a*, *b*, *c* (Å)	33.0258 (7), 5.4412 (1), 22.4342 (4)	5.6715 (1), 7.4164 (1), 15.9819 (3)
α, β, γ (°)	90, 107.203 (2), 90	88.369 (1), 84.147 (1), 82.961 (2)
*V* (Å^3^)	3851.07 (13)	663.60 (2)
*Z*	8	2
Radiation type	Mo *K*α	Cu *K*α
μ (mm^−1^)	0.16	0.84
Crystal size (mm)	0.40 × 0.08 × 0.04	0.22 × 0.12 × 0.05

Data collection		
Diffractometer	Rigaku FRE+ equipped with VHF Varimax confocal mirrors and an AFC12 goniometer and HyPix 6000 detector	Rigaku 007HF equipped with Varimax confocal mirrors and an AFC11 goniometer and HyPix 6000 detector
Absorption correction	Gaussian (*CrysAlis PRO*; Rigaku OD, 2019)	Multi-scan (*CrysAlis PRO*; Rigaku OD, 2019)
*T*_min_, *T*_max_	0.837, 1.000	0.930, 1.000
No. of measured, independent and observed [*I* > 2σ(*I*)] reflections	22915, 4381, 3983	11641, 2352, 2250
*R*_int_	0.016	0.027
(sin θ/λ)_max_ (Å^−1^)	0.649	0.597

Refinement		
*R*[*F* ^2^ > 2σ(*F* ^2^)], *wR*(*F* ^2^), *S*	0.032, 0.086, 1.05	0.033, 0.104, 0.88
No. of reflections	4381	2352
No. of parameters	298	203
H-atom treatment	H atoms treated by a mixture of independent and constrained refinement	H atoms treated by a mixture of independent and constrained refinement
Δρ_max_, Δρ_min_ (e Å^−3^)	0.31, −0.30	0.23, −0.19

Computer programs: *CrysAlis PRO* (Rigaku OD, 2019 ▸), *OSCAIL* (McArdle *et al.*, 2004 ▸), *SHELXT* (Sheldrick, 2015*a* ▸), *ShelXle* (Hübschle *et al.*, 2011 ▸) *SHELXL2017* (Sheldrick, 2015*b* ▸), *Mercury* (Macrae *et al.*, 2006 ▸), and *PLATON* (Spek, 2009 ▸).

## supplementary crystallographic information

### (E)-2-Oxo-N'-(3,4,5-trimethoxybenzylidene)-2H-chromene-3-carbohydrazide dimethyl sulfoxide hemisolvate (I) Crystal data

**Table:**

C_20_H_18_N_2_O_6_·0.5C_2_H_6_OS	*F*(000) = 1768
*M**_r_* = 421.43	*D*_x_ = 1.454 Mg m^−^^3^
Monoclinic, *C*2/*c*	Mo *K*α radiation, λ = 0.71075 Å
*a* = 33.0258 (7) Å	Cell parameters from 13857 reflections
*b* = 5.4412 (1) Å	θ = 2.0–31.6°
*c* = 22.4342 (4) Å	µ = 0.16 mm^−^^1^
β = 107.203 (2)°	*T* = 100 K
*V* = 3851.07 (13) Å^3^	Block, yellow
*Z* = 8	0.40 × 0.08 × 0.04 mm

### (E)-2-Oxo-N'-(3,4,5-trimethoxybenzylidene)-2H-chromene-3-carbohydrazide dimethyl sulfoxide hemisolvate (I) Data collection

**Table:**

Rigaku FRE+ equipped with VHF Varimax confocal mirrors and an AFC12 goniometer and HyPix 6000 detector diffractometer	4381 independent reflections
Radiation source: Rotating Anode, Rigaku FRE+	3983 reflections with *I* > 2σ(*I*)
Confocal mirrors, VHF Varimax monochromator	*R*_int_ = 0.016
Detector resolution: 10 pixels mm^-1^	θ_max_ = 27.5°, θ_min_ = 2.6°
profile data from ω–scans	*h* = −42→42
Absorption correction: gaussian (CrysAlisPro; Rigaku OD, 2019)	*k* = −6→7
*T*_min_ = 0.837, *T*_max_ = 1.000	*l* = −26→29
22915 measured reflections	

### (E)-2-Oxo-N'-(3,4,5-trimethoxybenzylidene)-2H-chromene-3-carbohydrazide dimethyl sulfoxide hemisolvate (I) Refinement

**Table:**

Refinement on *F*^2^	0 restraints
Least-squares matrix: full	Hydrogen site location: mixed
*R*[*F*^2^ > 2σ(*F*^2^)] = 0.032	H atoms treated by a mixture of independent and constrained refinement
*wR*(*F*^2^) = 0.086	*w* = 1/[σ^2^(*F*_o_^2^) + (0.0456*P*)^2^ + 2.9167*P*] where *P* = (*F*_o_^2^ + 2*F*_c_^2^)/3
*S* = 1.05	(Δ/σ)_max_ = 0.001
4381 reflections	Δρ_max_ = 0.31 e Å^−^^3^
298 parameters	Δρ_min_ = −0.30 e Å^−^^3^

### (E)-2-Oxo-N'-(3,4,5-trimethoxybenzylidene)-2H-chromene-3-carbohydrazide dimethyl sulfoxide hemisolvate (I) Special details

**Table:**

Geometry. All esds (except the esd in the dihedral angle between two l.s. planes) are estimated using the full covariance matrix. The cell esds are taken into account individually in the estimation of esds in distances, angles and torsion angles; correlations between esds in cell parameters are only used when they are defined by crystal symmetry. An approximate (isotropic) treatment of cell esds is used for estimating esds involving l.s. planes.

### (E)-2-Oxo-N'-(3,4,5-trimethoxybenzylidene)-2H-chromene-3-carbohydrazide dimethyl sulfoxide hemisolvate (I) Fractional atomic coordinates and isotropic or equivalent isotropic displacement parameters (Å^2^)

**Table:**

	*x*	*y*	*z*	*U*_iso_*/*U*_eq_	Occ. (<1)
O1	0.40457 (2)	0.68439 (13)	0.52398 (3)	0.01627 (16)	
O2	0.46021 (2)	0.81610 (14)	0.59671 (4)	0.02005 (17)	
O31	0.53178 (2)	0.20068 (14)	0.57120 (3)	0.01931 (17)	
O343	0.72470 (2)	0.31586 (14)	0.80720 (3)	0.01717 (16)	
O344	0.73938 (2)	0.64983 (14)	0.89856 (3)	0.01614 (16)	
O345	0.67832 (2)	0.95654 (15)	0.90901 (3)	0.02104 (17)	
N32	0.53224 (3)	0.55236 (18)	0.62643 (4)	0.01798 (19)	
H32	0.5171 (5)	0.679 (3)	0.6305 (7)	0.029 (4)*	
N33	0.57200 (3)	0.51012 (17)	0.66718 (4)	0.01668 (18)	
C2	0.44611 (3)	0.65614 (18)	0.55853 (4)	0.01415 (19)	
C3	0.46844 (3)	0.43809 (18)	0.54634 (4)	0.01325 (19)	
C4	0.44791 (3)	0.27552 (19)	0.50194 (4)	0.01369 (19)	
H4	0.462640	0.135045	0.494099	0.016*	
C5	0.38134 (3)	0.14563 (19)	0.42087 (5)	0.0175 (2)	
H5	0.394775	0.003909	0.410755	0.021*	
C4A	0.40442 (3)	0.31084 (18)	0.46651 (4)	0.01345 (19)	
C6	0.33896 (3)	0.1903 (2)	0.39072 (5)	0.0204 (2)	
H6	0.323245	0.077292	0.360352	0.024*	
C7	0.31911 (3)	0.4001 (2)	0.40457 (5)	0.0194 (2)	
H7	0.289997	0.428309	0.383493	0.023*	
C8	0.34135 (3)	0.5677 (2)	0.44868 (5)	0.0173 (2)	
H8	0.328020	0.711508	0.457860	0.021*	
C8A	0.38373 (3)	0.51893 (18)	0.47906 (4)	0.01401 (19)	
C31	0.51385 (3)	0.38442 (19)	0.58215 (4)	0.0144 (2)	
C34	0.58354 (3)	0.6722 (2)	0.70991 (5)	0.0234 (2)	
H34	0.565334	0.807515	0.709773	0.028*	
C341	0.62400 (3)	0.6560 (2)	0.75915 (5)	0.0182 (2)	
C342	0.65468 (3)	0.48341 (19)	0.75655 (4)	0.0154 (2)	
H342	0.649709	0.371929	0.722555	0.018*	
C343	0.69263 (3)	0.47723 (18)	0.80448 (4)	0.01379 (19)	
C344	0.70034 (3)	0.64402 (19)	0.85427 (4)	0.0140 (2)	
C345	0.66880 (3)	0.80974 (19)	0.85746 (5)	0.0165 (2)	
C346	0.63073 (3)	0.8169 (2)	0.80957 (5)	0.0207 (2)	
H346	0.609346	0.931306	0.811211	0.025*	
C431	0.71732 (4)	0.1389 (2)	0.75813 (5)	0.0207 (2)	
H43A	0.712251	0.223812	0.718061	0.031*	
H43B	0.692471	0.039486	0.757575	0.031*	
H43C	0.742175	0.032094	0.765084	0.031*	
C441	0.74230 (3)	0.4860 (2)	0.94982 (5)	0.0195 (2)	
H41A	0.736607	0.317514	0.934155	0.029*	
H41B	0.721429	0.533664	0.970960	0.029*	
H41C	0.770842	0.495051	0.979338	0.029*	
C451	0.64624 (3)	1.1251 (2)	0.91407 (5)	0.0196 (2)	
H51A	0.620280	1.034335	0.912316	0.029*	
H51B	0.640421	1.242953	0.879538	0.029*	
H51C	0.656046	1.213390	0.953827	0.029*	
S1S	0.50726 (2)	1.12167 (9)	0.72755 (2)	0.01665 (11)	0.5
O1S	0.4985 (16)	0.8678 (3)	0.7444 (12)	0.026 (2)	0.5
C1S	0.45886 (14)	1.2893 (14)	0.7013 (3)	0.0207 (9)	0.5
H1SA	0.442339	1.228493	0.660195	0.031*	0.5
H1SB	0.465103	1.464184	0.698321	0.031*	0.5
H1SC	0.442536	1.267478	0.730985	0.031*	0.5
C2S	0.52800 (15)	1.2784 (15)	0.7996 (3)	0.0300 (12)	0.5
H2SA	0.510606	1.241255	0.827053	0.045*	0.5
H2SB	0.527643	1.455833	0.791972	0.045*	0.5
H2SC	0.557203	1.224526	0.819443	0.045*	0.5

### (E)-2-Oxo-N'-(3,4,5-trimethoxybenzylidene)-2H-chromene-3-carbohydrazide dimethyl sulfoxide hemisolvate (I) Atomic displacement parameters (Å^2^)

**Table:**

	*U*^11^	*U*^22^	*U*^33^	*U*^12^	*U*^13^	*U*^23^
O1	0.0125 (3)	0.0167 (4)	0.0169 (3)	0.0020 (3)	0.0002 (3)	−0.0032 (3)
O2	0.0179 (4)	0.0181 (4)	0.0202 (4)	0.0021 (3)	−0.0004 (3)	−0.0066 (3)
O31	0.0143 (3)	0.0207 (4)	0.0198 (4)	0.0040 (3)	0.0002 (3)	−0.0052 (3)
O343	0.0150 (3)	0.0195 (4)	0.0151 (3)	0.0036 (3)	0.0015 (3)	−0.0017 (3)
O344	0.0122 (3)	0.0200 (4)	0.0129 (3)	−0.0033 (3)	−0.0014 (3)	0.0018 (3)
O345	0.0171 (4)	0.0253 (4)	0.0179 (4)	0.0004 (3)	0.0008 (3)	−0.0105 (3)
N32	0.0103 (4)	0.0222 (5)	0.0177 (4)	0.0040 (3)	−0.0017 (3)	−0.0063 (3)
N33	0.0104 (4)	0.0231 (5)	0.0144 (4)	0.0001 (3)	0.0003 (3)	−0.0022 (3)
C2	0.0122 (4)	0.0160 (5)	0.0130 (4)	0.0007 (4)	0.0018 (3)	0.0001 (4)
C3	0.0115 (4)	0.0149 (5)	0.0124 (4)	0.0008 (4)	0.0022 (3)	0.0002 (4)
C4	0.0135 (4)	0.0140 (5)	0.0130 (4)	0.0007 (4)	0.0031 (4)	0.0006 (4)
C5	0.0183 (5)	0.0165 (5)	0.0156 (5)	−0.0020 (4)	0.0018 (4)	−0.0006 (4)
C4A	0.0129 (4)	0.0147 (5)	0.0117 (4)	−0.0013 (4)	0.0021 (3)	0.0015 (3)
C6	0.0189 (5)	0.0223 (5)	0.0155 (5)	−0.0060 (4)	−0.0020 (4)	−0.0002 (4)
C7	0.0124 (5)	0.0248 (6)	0.0171 (5)	−0.0022 (4)	−0.0015 (4)	0.0060 (4)
C8	0.0139 (5)	0.0186 (5)	0.0183 (5)	0.0016 (4)	0.0031 (4)	0.0042 (4)
C8A	0.0135 (4)	0.0148 (5)	0.0125 (4)	−0.0021 (4)	0.0020 (4)	0.0008 (4)
C31	0.0119 (4)	0.0179 (5)	0.0123 (4)	0.0003 (4)	0.0019 (3)	−0.0009 (4)
C34	0.0143 (5)	0.0289 (6)	0.0226 (5)	0.0051 (4)	−0.0011 (4)	−0.0099 (4)
C341	0.0131 (5)	0.0229 (5)	0.0163 (5)	−0.0007 (4)	0.0008 (4)	−0.0050 (4)
C342	0.0142 (4)	0.0188 (5)	0.0122 (4)	−0.0016 (4)	0.0024 (4)	−0.0034 (4)
C343	0.0132 (4)	0.0151 (5)	0.0136 (4)	−0.0005 (4)	0.0047 (4)	0.0014 (4)
C344	0.0109 (4)	0.0177 (5)	0.0116 (4)	−0.0032 (4)	0.0007 (3)	0.0010 (4)
C345	0.0157 (5)	0.0191 (5)	0.0137 (5)	−0.0032 (4)	0.0031 (4)	−0.0049 (4)
C346	0.0140 (5)	0.0250 (6)	0.0209 (5)	0.0029 (4)	0.0019 (4)	−0.0081 (4)
C431	0.0224 (5)	0.0185 (5)	0.0203 (5)	0.0032 (4)	0.0051 (4)	−0.0037 (4)
C441	0.0193 (5)	0.0220 (5)	0.0145 (5)	−0.0009 (4)	0.0007 (4)	0.0032 (4)
C451	0.0180 (5)	0.0209 (5)	0.0205 (5)	−0.0021 (4)	0.0064 (4)	−0.0075 (4)
S1S	0.0182 (2)	0.0149 (2)	0.0183 (2)	0.00064 (18)	0.00770 (19)	−0.00093 (19)
O1S	0.040 (5)	0.0140 (6)	0.030 (7)	0.0000 (17)	0.020 (7)	−0.0005 (12)
C1S	0.019 (2)	0.0187 (14)	0.0218 (14)	0.003 (2)	0.0018 (17)	0.0027 (10)
C2S	0.033 (3)	0.024 (2)	0.0284 (17)	0.006 (3)	0.001 (2)	−0.0054 (13)

### (E)-2-Oxo-N'-(3,4,5-trimethoxybenzylidene)-2H-chromene-3-carbohydrazide dimethyl sulfoxide hemisolvate (I) Geometric parameters (Å, º)

**Table:**

O1—C2	1.3702 (12)	C34—C341	1.4629 (14)
O1—C8A	1.3747 (12)	C34—H34	0.9500
O2—C2	1.2133 (12)	C341—C342	1.3953 (14)
O31—C31	1.2234 (13)	C341—C346	1.3955 (14)
O343—C343	1.3631 (12)	C342—C343	1.3894 (13)
O343—C431	1.4280 (12)	C342—H342	0.9500
O344—C344	1.3759 (11)	C343—C344	1.4028 (14)
O344—C441	1.4356 (12)	C344—C345	1.3955 (14)
O345—C345	1.3635 (12)	C345—C346	1.3921 (14)
O345—C451	1.4309 (13)	C346—H346	0.9500
N32—C31	1.3543 (13)	C431—H43A	0.9800
N32—N33	1.3793 (11)	C431—H43B	0.9800
N32—H32	0.870 (16)	C431—H43C	0.9800
N33—C34	1.2753 (14)	C441—H41A	0.9800
C2—C3	1.4647 (13)	C441—H41B	0.9800
C3—C4	1.3547 (14)	C441—H41C	0.9800
C3—C31	1.5056 (13)	C451—H51A	0.9800
C4—C4A	1.4340 (13)	C451—H51B	0.9800
C4—H4	0.9500	C451—H51C	0.9800
C5—C6	1.3840 (15)	S1S—O1S	1.483 (16)
C5—C4A	1.4057 (14)	S1S—C2S	1.775 (7)
C5—H5	0.9500	S1S—C1S	1.782 (5)
C4A—C8A	1.3935 (14)	C1S—H1SA	0.9800
C6—C7	1.3965 (16)	C1S—H1SB	0.9800
C6—H6	0.9500	C1S—H1SC	0.9800
C7—C8	1.3856 (15)	C2S—H2SA	0.9800
C7—H7	0.9500	C2S—H2SB	0.9800
C8—C8A	1.3890 (13)	C2S—H2SC	0.9800
C8—H8	0.9500		
			
C2—O1—C8A	122.80 (8)	C341—C342—H342	120.5
C343—O343—C431	116.52 (8)	O343—C343—C342	124.14 (9)
C344—O344—C441	113.00 (8)	O343—C343—C344	115.17 (8)
C345—O345—C451	116.90 (8)	C342—C343—C344	120.69 (9)
C31—N32—N33	120.41 (9)	O344—C344—C345	120.16 (9)
C31—N32—H32	117.6 (10)	O344—C344—C343	120.05 (9)
N33—N32—H32	121.7 (10)	C345—C344—C343	119.76 (9)
C34—N33—N32	113.41 (9)	O345—C345—C346	124.54 (9)
O2—C2—O1	115.46 (9)	O345—C345—C344	115.71 (9)
O2—C2—C3	127.12 (9)	C346—C345—C344	119.75 (9)
O1—C2—C3	117.42 (8)	C345—C346—C341	119.92 (10)
C4—C3—C2	119.74 (9)	C345—C346—H346	120.0
C4—C3—C31	117.95 (9)	C341—C346—H346	120.0
C2—C3—C31	122.31 (9)	O343—C431—H43A	109.5
C3—C4—C4A	121.44 (9)	O343—C431—H43B	109.5
C3—C4—H4	119.3	H43A—C431—H43B	109.5
C4A—C4—H4	119.3	O343—C431—H43C	109.5
C6—C5—C4A	119.72 (10)	H43A—C431—H43C	109.5
C6—C5—H5	120.1	H43B—C431—H43C	109.5
C4A—C5—H5	120.1	O344—C441—H41A	109.5
C8A—C4A—C5	118.30 (9)	O344—C441—H41B	109.5
C8A—C4A—C4	117.88 (9)	H41A—C441—H41B	109.5
C5—C4A—C4	123.81 (9)	O344—C441—H41C	109.5
C5—C6—C7	120.56 (10)	H41A—C441—H41C	109.5
C5—C6—H6	119.7	H41B—C441—H41C	109.5
C7—C6—H6	119.7	O345—C451—H51A	109.5
C8—C7—C6	120.80 (9)	O345—C451—H51B	109.5
C8—C7—H7	119.6	H51A—C451—H51B	109.5
C6—C7—H7	119.6	O345—C451—H51C	109.5
C7—C8—C8A	117.99 (10)	H51A—C451—H51C	109.5
C7—C8—H8	121.0	H51B—C451—H51C	109.5
C8A—C8—H8	121.0	O1S—S1S—C2S	105.5 (10)
O1—C8A—C8	116.63 (9)	O1S—S1S—C1S	109.8 (19)
O1—C8A—C4A	120.73 (9)	C2S—S1S—C1S	96.99 (18)
C8—C8A—C4A	122.62 (9)	S1S—C1S—H1SA	109.5
O31—C31—N32	124.05 (9)	S1S—C1S—H1SB	109.5
O31—C31—C3	121.09 (9)	H1SA—C1S—H1SB	109.5
N32—C31—C3	114.86 (9)	S1S—C1S—H1SC	109.5
N33—C34—C341	121.94 (10)	H1SA—C1S—H1SC	109.5
N33—C34—H34	119.0	H1SB—C1S—H1SC	109.5
C341—C34—H34	119.0	S1S—C2S—H2SA	109.5
C342—C341—C346	120.85 (9)	S1S—C2S—H2SB	109.5
C342—C341—C34	121.44 (9)	H2SA—C2S—H2SB	109.5
C346—C341—C34	117.71 (9)	S1S—C2S—H2SC	109.5
C343—C342—C341	118.94 (9)	H2SA—C2S—H2SC	109.5
C343—C342—H342	120.5	H2SB—C2S—H2SC	109.5
			
C31—N32—N33—C34	−173.67 (10)	C4—C3—C31—N32	−179.03 (9)
C8A—O1—C2—O2	−179.61 (9)	C2—C3—C31—N32	0.25 (14)
C8A—O1—C2—C3	−0.32 (13)	N32—N33—C34—C341	177.68 (10)
O2—C2—C3—C4	179.69 (10)	N33—C34—C341—C342	9.81 (18)
O1—C2—C3—C4	0.50 (14)	N33—C34—C341—C346	−169.23 (11)
O2—C2—C3—C31	0.42 (16)	C346—C341—C342—C343	−1.33 (16)
O1—C2—C3—C31	−178.77 (8)	C34—C341—C342—C343	179.66 (10)
C2—C3—C4—C4A	−0.40 (14)	C431—O343—C343—C342	−2.19 (14)
C31—C3—C4—C4A	178.90 (9)	C431—O343—C343—C344	178.22 (9)
C6—C5—C4A—C8A	−1.25 (15)	C341—C342—C343—O343	179.45 (9)
C6—C5—C4A—C4	177.48 (9)	C341—C342—C343—C344	−0.98 (15)
C3—C4—C4A—C8A	0.11 (14)	C441—O344—C344—C345	92.74 (11)
C3—C4—C4A—C5	−178.62 (9)	C441—O344—C344—C343	−89.56 (11)
C4A—C5—C6—C7	1.01 (16)	O343—C343—C344—O344	5.20 (13)
C5—C6—C7—C8	0.00 (16)	C342—C343—C344—O344	−174.41 (9)
C6—C7—C8—C8A	−0.72 (15)	O343—C343—C344—C345	−177.09 (9)
C2—O1—C8A—C8	178.49 (9)	C342—C343—C344—C345	3.30 (15)
C2—O1—C8A—C4A	0.04 (14)	C451—O345—C345—C346	1.34 (15)
C7—C8—C8A—O1	−177.96 (9)	C451—O345—C345—C344	−178.86 (9)
C7—C8—C8A—C4A	0.46 (15)	O344—C344—C345—O345	−5.41 (14)
C5—C4A—C8A—O1	178.88 (9)	C343—C344—C345—O345	176.88 (9)
C4—C4A—C8A—O1	0.08 (14)	O344—C344—C345—C346	174.40 (9)
C5—C4A—C8A—C8	0.52 (15)	C343—C344—C345—C346	−3.31 (15)
C4—C4A—C8A—C8	−178.28 (9)	O345—C345—C346—C341	−179.17 (10)
N33—N32—C31—O31	−7.04 (16)	C344—C345—C346—C341	1.03 (17)
N33—N32—C31—C3	172.52 (8)	C342—C341—C346—C345	1.31 (17)
C4—C3—C31—O31	0.54 (14)	C34—C341—C346—C345	−179.64 (10)
C2—C3—C31—O31	179.82 (9)		

### (E)-2-Oxo-N'-(3,4,5-trimethoxybenzylidene)-2H-chromene-3-carbohydrazide dimethyl sulfoxide hemisolvate (I) Hydrogen-bond geometry (Å, º)

*Cg*1, *Cg*2 and *Cg*3 are the centroids of the O1/C2–C4/C4*A*/C8*A*, C4*A*/C5–C8/C8*A* and C341–C346 rings, respectively.

**Table:**

*D*—H···*A*	*D*—H	H···*A*	*D*···*A*	*D*—H···*A*
N32—H32···O2	0.870 (16)	1.955 (15)	2.6878 (12)	141.0 (14)
C441—H41*C*···O345^i^	0.98	2.58	3.4772 (12)	152
C451—H51*A*···O1^ii^	0.98	2.65	3.4463 (13)	138
C34—H34···O1*S*	0.95	2.57	3.30 (5)	134
C34—H34···O1*S*^ii^	0.95	2.63	3.34 (5)	133
C34—H34···S1*S*	0.95	2.69	3.6158 (12)	166
C431—H43*C*···O343^iii^	0.98	2.50	3.2505 (13)	133
C2*S*—H2*SA*···N32^iv^	0.98	2.61	3.3000 (6)	127
C4—H4···O31	0.95	2.45	2.7761 (12)	100
C4—H4···O31^v^	0.95	2.38	3.2415 (12)	150
C5—H5···O31^v^	0.95	2.59	3.3931 (13)	143
C431—H43*B*···*Cg*3^vi^	0.98	2.73	3.5882 (13)	147
C451—H51*B*···*Cg*3^vi^	0.98	2.95	3.8562 (12)	155
C451—H51*C*···*Cg*2^vii^	0.98	2.83	3.6883 (13)	147
C31—O31···*Cg*1^vii^	0	0	3.3971 (6)	90 (1)

Symmetry codes: (i) −*x*+3/2, −*y*+3/2, −*z*+2; (ii) −*x*+1, *y*, −*z*+3/2; (iii) −*x*+3/2, *y*−1/2, −*z*+3/2; (iv) −*x*+1, *y*+1, −*z*+3/2; (v) −*x*+1, −*y*, −*z*+1; (vi) *x*, *y*−1, *z*; (vii) −*x*+1, −*y*+1, −*z*+1.

### (E)-N'-Benzylidene-2-oxo-2H-chromene-3-carbohydrazide (II) Crystal data

**Table:**

C_17_H_12_N_2_O_3_	*Z* = 2
*M**_r_* = 292.29	*F*(000) = 304
Triclinic, *P*1	*D*_x_ = 1.463 Mg m^−^^3^
*a* = 5.6715 (1) Å	Cu *K*α radiation, λ = 1.54178 Å
*b* = 7.4164 (1) Å	Cell parameters from 8290 reflections
*c* = 15.9819 (3) Å	θ = 6.0–70.3°
α = 88.369 (1)°	µ = 0.84 mm^−^^1^
β = 84.147 (1)°	*T* = 100 K
γ = 82.961 (2)°	Plate, colourless
*V* = 663.60 (2) Å^3^	0.22 × 0.12 × 0.05 mm

### (E)-N'-Benzylidene-2-oxo-2H-chromene-3-carbohydrazide (II) Data collection

**Table:**

Rigaku 007HF equipped with Varimax confocal mirrors and an AFC11 goniometer and HyPix 6000 detector diffractometer	2352 independent reflections
Radiation source: Rotating anode, Rigaku 007 HF	2250 reflections with *I* > 2σ(*I*)
Varimax focusing mirrors monochromator	*R*_int_ = 0.027
Detector resolution: 10 pixels mm^-1^	θ_max_ = 67.1°, θ_min_ = 5.6°
profile data from ω–scans	*h* = −6→6
Absorption correction: multi-scan (CrysAlisPro; Rigaku OD, 2019)	*k* = −8→8
*T*_min_ = 0.930, *T*_max_ = 1.000	*l* = −19→19
11641 measured reflections	

### (E)-N'-Benzylidene-2-oxo-2H-chromene-3-carbohydrazide (II) Refinement

**Table:**

Refinement on *F*^2^	0 restraints
Least-squares matrix: full	Hydrogen site location: mixed
*R*[*F*^2^ > 2σ(*F*^2^)] = 0.033	H atoms treated by a mixture of independent and constrained refinement
*wR*(*F*^2^) = 0.104	*w* = 1/[σ^2^(*F*_o_^2^) + (0.0858*P*)^2^ + 0.127*P*] where *P* = (*F*_o_^2^ + 2*F*_c_^2^)/3
*S* = 0.88	(Δ/σ)_max_ = 0.001
2352 reflections	Δρ_max_ = 0.23 e Å^−^^3^
203 parameters	Δρ_min_ = −0.19 e Å^−^^3^

### (E)-N'-Benzylidene-2-oxo-2H-chromene-3-carbohydrazide (II) Special details

**Table:**

Geometry. All esds (except the esd in the dihedral angle between two l.s. planes) are estimated using the full covariance matrix. The cell esds are taken into account individually in the estimation of esds in distances, angles and torsion angles; correlations between esds in cell parameters are only used when they are defined by crystal symmetry. An approximate (isotropic) treatment of cell esds is used for estimating esds involving l.s. planes.

### (E)-N'-Benzylidene-2-oxo-2H-chromene-3-carbohydrazide (II) Fractional atomic coordinates and isotropic or equivalent isotropic displacement parameters (Å^2^)

**Table:**

	*x*	*y*	*z*	*U*_iso_*/*U*_eq_	
O1	0.38486 (11)	0.36429 (9)	0.68964 (4)	0.0233 (2)	
H1	0.276 (3)	0.3194 (18)	0.4545 (9)	0.043 (4)*	
O2	0.19764 (11)	0.41255 (9)	0.57568 (4)	0.0256 (2)	
O31	0.77445 (12)	0.11739 (10)	0.43121 (4)	0.0316 (2)	
N32	0.40134 (15)	0.26503 (11)	0.42751 (5)	0.0220 (2)	
N33	0.40966 (14)	0.23814 (10)	0.34233 (5)	0.0227 (2)	
C2	0.37588 (16)	0.34215 (12)	0.60485 (6)	0.0213 (2)	
C3	0.58217 (17)	0.23662 (12)	0.55958 (6)	0.0213 (2)	
C4	0.76971 (17)	0.16687 (12)	0.60074 (6)	0.0223 (2)	
H4	0.9026	0.0993	0.5703	0.027*	
C5	0.96435 (17)	0.12171 (13)	0.73471 (6)	0.0240 (2)	
H5	1.1005	0.0524	0.7070	0.029*	
C4A	0.77384 (17)	0.19200 (12)	0.68915 (6)	0.0217 (2)	
C6	0.95376 (17)	0.15324 (13)	0.81971 (6)	0.0256 (2)	
H6	1.0835	0.1068	0.8504	0.031*	
C7	0.75272 (18)	0.25331 (13)	0.86075 (6)	0.0264 (2)	
H7	0.7472	0.2744	0.9193	0.032*	
C8	0.56165 (18)	0.32215 (13)	0.81749 (6)	0.0251 (2)	
H8	0.4243	0.3888	0.8458	0.030*	
C8A	0.57482 (17)	0.29175 (12)	0.73184 (6)	0.0219 (2)	
C31	0.59517 (17)	0.20132 (12)	0.46715 (6)	0.0232 (2)	
C34	0.22002 (17)	0.29462 (12)	0.30817 (6)	0.0216 (2)	
H34	0.0821	0.3485	0.3413	0.026*	
C341	0.21746 (16)	0.27545 (12)	0.21730 (6)	0.0215 (2)	
C342	0.41939 (17)	0.19665 (13)	0.16727 (6)	0.0238 (2)	
H342	0.5605	0.1523	0.1923	0.029*	
C343	0.41260 (17)	0.18368 (13)	0.08144 (6)	0.0274 (2)	
H343	0.5495	0.1299	0.0478	0.033*	
C344	0.20758 (19)	0.24849 (14)	0.04380 (6)	0.0289 (2)	
H344	0.2048	0.2399	−0.0153	0.035*	
C345	0.00689 (18)	0.32579 (14)	0.09316 (6)	0.0280 (2)	
H345	−0.1337	0.3703	0.0678	0.034*	
C346	0.01167 (17)	0.33801 (13)	0.17960 (6)	0.0249 (2)	
H346	−0.1267	0.3895	0.2132	0.030*	

### (E)-N'-Benzylidene-2-oxo-2H-chromene-3-carbohydrazide (II) Atomic displacement parameters (Å^2^)

**Table:**

	*U*^11^	*U*^22^	*U*^33^	*U*^12^	*U*^13^	*U*^23^
O1	0.0226 (4)	0.0284 (4)	0.0176 (4)	0.0025 (3)	−0.0017 (3)	−0.0030 (3)
O2	0.0214 (4)	0.0315 (4)	0.0223 (4)	0.0050 (3)	−0.0029 (3)	−0.0030 (3)
O31	0.0276 (4)	0.0432 (4)	0.0196 (4)	0.0139 (3)	−0.0021 (3)	−0.0048 (3)
N32	0.0215 (4)	0.0275 (4)	0.0155 (4)	0.0034 (3)	−0.0008 (3)	−0.0034 (3)
N33	0.0251 (4)	0.0257 (4)	0.0165 (4)	0.0002 (3)	−0.0014 (3)	−0.0022 (3)
C2	0.0229 (5)	0.0227 (5)	0.0181 (5)	−0.0016 (4)	−0.0014 (4)	−0.0013 (3)
C3	0.0215 (5)	0.0223 (5)	0.0192 (5)	−0.0002 (4)	−0.0009 (4)	−0.0011 (4)
C4	0.0226 (5)	0.0231 (5)	0.0201 (5)	−0.0001 (4)	0.0005 (4)	−0.0017 (4)
C5	0.0245 (5)	0.0251 (5)	0.0224 (5)	−0.0026 (4)	−0.0024 (4)	−0.0004 (4)
C4A	0.0236 (5)	0.0218 (5)	0.0199 (5)	−0.0032 (4)	−0.0023 (4)	−0.0002 (3)
C6	0.0279 (5)	0.0270 (5)	0.0232 (5)	−0.0045 (4)	−0.0076 (4)	0.0016 (4)
C7	0.0348 (5)	0.0279 (5)	0.0175 (5)	−0.0066 (4)	−0.0037 (4)	−0.0015 (4)
C8	0.0285 (5)	0.0260 (5)	0.0200 (5)	−0.0021 (4)	0.0004 (4)	−0.0029 (4)
C8A	0.0238 (5)	0.0221 (5)	0.0201 (5)	−0.0031 (4)	−0.0031 (4)	−0.0001 (4)
C31	0.0237 (5)	0.0242 (5)	0.0200 (5)	0.0021 (4)	−0.0006 (4)	−0.0009 (4)
C34	0.0204 (5)	0.0227 (5)	0.0208 (5)	0.0009 (3)	−0.0008 (4)	−0.0013 (3)
C341	0.0233 (5)	0.0212 (5)	0.0200 (5)	−0.0025 (4)	−0.0024 (4)	−0.0008 (4)
C342	0.0218 (5)	0.0270 (5)	0.0222 (5)	−0.0007 (4)	−0.0033 (4)	−0.0009 (4)
C343	0.0272 (5)	0.0315 (5)	0.0225 (5)	−0.0025 (4)	0.0022 (4)	−0.0036 (4)
C344	0.0360 (6)	0.0336 (5)	0.0178 (5)	−0.0057 (4)	−0.0040 (4)	−0.0014 (4)
C345	0.0288 (5)	0.0310 (5)	0.0247 (5)	−0.0011 (4)	−0.0089 (4)	0.0005 (4)
C346	0.0236 (5)	0.0262 (5)	0.0239 (5)	0.0013 (4)	−0.0028 (4)	−0.0015 (4)

### (E)-N'-Benzylidene-2-oxo-2H-chromene-3-carbohydrazide (II) Geometric parameters (Å, º)

**Table:**

O1—C8A	1.3749 (11)	C6—H6	0.9500
O1—C2	1.3765 (11)	C7—C8	1.3820 (14)
O2—C2	1.2103 (11)	C7—H7	0.9500
O31—C31	1.2237 (12)	C8—C8A	1.3867 (13)
N32—C31	1.3530 (13)	C8—H8	0.9500
N32—N33	1.3768 (11)	C34—C341	1.4649 (13)
N32—H1	0.857 (15)	C34—H34	0.9500
N33—C34	1.2753 (13)	C341—C346	1.3912 (13)
C2—C3	1.4629 (13)	C341—C342	1.4030 (13)
C3—C4	1.3492 (13)	C342—C343	1.3826 (13)
C3—C31	1.5003 (13)	C342—H342	0.9500
C4—C4A	1.4334 (13)	C343—C344	1.3908 (14)
C4—H4	0.9500	C343—H343	0.9500
C5—C6	1.3790 (13)	C344—C345	1.3890 (15)
C5—C4A	1.4036 (13)	C344—H344	0.9500
C5—H5	0.9500	C345—C346	1.3903 (13)
C4A—C8A	1.3978 (14)	C345—H345	0.9500
C6—C7	1.3960 (14)	C346—H346	0.9500
			
C8A—O1—C2	123.10 (7)	C8A—C8—H8	120.7
C31—N32—N33	118.22 (8)	O1—C8A—C8	117.65 (8)
C31—N32—H1	121.3 (9)	O1—C8A—C4A	120.69 (8)
N33—N32—H1	120.5 (9)	C8—C8A—C4A	121.66 (9)
C34—N33—N32	116.05 (8)	O31—C31—N32	123.05 (9)
O2—C2—O1	116.32 (8)	O31—C31—C3	120.11 (9)
O2—C2—C3	126.92 (8)	N32—C31—C3	116.83 (8)
O1—C2—C3	116.76 (8)	N33—C34—C341	119.21 (8)
C4—C3—C2	120.21 (9)	N33—C34—H34	120.4
C4—C3—C31	117.51 (8)	C341—C34—H34	120.4
C2—C3—C31	122.28 (8)	C346—C341—C342	119.23 (9)
C3—C4—C4A	121.77 (9)	C346—C341—C34	119.47 (8)
C3—C4—H4	119.1	C342—C341—C34	121.30 (9)
C4A—C4—H4	119.1	C343—C342—C341	119.88 (9)
C6—C5—C4A	119.97 (9)	C343—C342—H342	120.1
C6—C5—H5	120.0	C341—C342—H342	120.1
C4A—C5—H5	120.0	C342—C343—C344	120.75 (9)
C5—C4A—C8A	118.69 (9)	C342—C343—H343	119.6
C5—C4A—C4	123.84 (9)	C344—C343—H343	119.6
C8A—C4A—C4	117.47 (9)	C345—C344—C343	119.56 (9)
C5—C6—C7	120.12 (9)	C345—C344—H344	120.2
C5—C6—H6	119.9	C343—C344—H344	120.2
C7—C6—H6	119.9	C344—C345—C346	120.04 (9)
C8—C7—C6	121.01 (9)	C344—C345—H345	120.0
C8—C7—H7	119.5	C346—C345—H345	120.0
C6—C7—H7	119.5	C341—C346—C345	120.54 (9)
C7—C8—C8A	118.53 (9)	C341—C346—H346	119.7
C7—C8—H8	120.7	C345—C346—H346	119.7
			
C31—N32—N33—C34	177.57 (7)	C4—C4A—C8A—O1	−0.84 (14)
C8A—O1—C2—O2	179.69 (7)	C5—C4A—C8A—C8	−0.11 (14)
C8A—O1—C2—C3	−0.40 (13)	C4—C4A—C8A—C8	179.54 (8)
O2—C2—C3—C4	179.63 (9)	N33—N32—C31—O31	−1.83 (15)
O1—C2—C3—C4	−0.27 (13)	N33—N32—C31—C3	178.66 (7)
O2—C2—C3—C31	−0.41 (16)	C4—C3—C31—O31	−2.44 (14)
O1—C2—C3—C31	179.69 (7)	C2—C3—C31—O31	177.61 (9)
C2—C3—C4—C4A	0.36 (15)	C4—C3—C31—N32	177.08 (7)
C31—C3—C4—C4A	−179.59 (7)	C2—C3—C31—N32	−2.87 (14)
C6—C5—C4A—C8A	−0.67 (14)	N32—N33—C34—C341	178.28 (7)
C6—C5—C4A—C4	179.70 (8)	N33—C34—C341—C346	−179.53 (8)
C3—C4—C4A—C5	179.82 (8)	N33—C34—C341—C342	−0.13 (14)
C3—C4—C4A—C8A	0.18 (15)	C346—C341—C342—C343	0.63 (14)
C4A—C5—C6—C7	0.70 (14)	C34—C341—C342—C343	−178.78 (8)
C5—C6—C7—C8	0.06 (14)	C341—C342—C343—C344	0.20 (15)
C6—C7—C8—C8A	−0.82 (14)	C342—C343—C344—C345	−0.51 (15)
C2—O1—C8A—C8	−179.39 (7)	C343—C344—C345—C346	−0.01 (15)
C2—O1—C8A—C4A	0.97 (14)	C342—C341—C346—C345	−1.15 (14)
C7—C8—C8A—O1	−178.79 (7)	C34—C341—C346—C345	178.27 (8)
C7—C8—C8A—C4A	0.85 (15)	C344—C345—C346—C341	0.85 (15)
C5—C4A—C8A—O1	179.51 (8)		

### (E)-N'-Benzylidene-2-oxo-2H-chromene-3-carbohydrazide (II) Hydrogen-bond geometry (Å, º)

**Table:**

*D*—H···*A*	*D*—H	H···*A*	*D*···*A*	*D*—H···*A*
N32—H1···O2	0.857 (15)	2.062 (15)	2.7238 (10)	133.5 (12)
C34—H34···O2^i^	0.95	2.54	3.4417 (11)	159
C4—H4···O31	0.95	2.40	2.7415 (11)	101
C4—H4···O31^ii^	0.95	2.28	3.1377 (12)	149
C5—H5···O31^ii^	0.95	2.57	3.3456 (12)	139
C346—H346···O1^i^	0.95	2.63	3.5195 (11)	156

Symmetry codes: (i) −*x*, −*y*+1, −*z*+1; (ii) −*x*+2, −*y*, −*z*+1.
