# Crystal structures and Hirshfeld surface analyses of (*E*)-*N*′-benzyl­idene-2-oxo-2*H*-chromene-3-carbo­hydrazide and the disordered hemi-DMSO solvate of (*E*)-2-oxo-*N*′-(3,4,5-trimeth­oxybenzyl­idene)-2*H*-chromene-3-carbohydrazide: lattice energy and inter­molecular inter­action energy calculations for the former. Corrigendum

**DOI:** 10.1107/S2056989019014890

**Published:** 2019-11-29

**Authors:** Ligia R. Gomes, John Nicolson Low, James L. Wardell, Camila Capelini, José Daniel Figueroa Villar, Vitoria R.F. Câmara, Edson F. da Silva, Samir A. Carvalho

**Affiliations:** a REQUIMTE, Departamento de Química e Bioquímica, Faculdade de Ciências da Universidade do Porto, Rua do Campo Alegre, 687, P-4169-007, Porto, Portugal; b FP-ENAS-Faculdade de Ciências de Saúde, Escola Superior de Saúde da UFP, Universidade Fernando Pessoa, Rua Carlos da Maia, 296, P-4200-150, Porto, Portugal; c Department of Chemistry, University of Aberdeen, Meston Walk, Old Aberdeen, AB24 3UE, Scotland; d Instituto de Tecnologia em Fármacos – Farmanguinhos, Fundaçâo Oswaldo Cruz, 21041-250 Rio de Janeiro, RJ, Brazil; e Grupo de Química Medicinal, Departamento de Química, Instituto Militar de Engenharia, Praća General Tibúrcio 80, 22290-270 Rio de Janeiro, Brazil; f Escola de Ciéncia e Tecnologia – ECT, Universidade do Grande Rio – Unigranrio, 25071-202 Duque de Caxias, RJ, Brazil

## Abstract

In the paper by Gomes *et al.* [*Acta Cryst.* (2019), E**75**, 1403–1410], there was an error and omission in the author and affiliation list.

## Abstract 2

Erratum to *Acta Cryst.* (2019), E**75**, 1403–1410.

In the paper by Gomes *et al.* (2019 ▸), one author was omitted (*i.e.* José Daniel Figueroa Villar) and the name and affiliations of another author (*i.e.*Camila Capelini) have been corrected. The complete and correct author list is given above.
